# A nonsynonymous mutation in *PLCG2* reduces the risk of Alzheimer’s disease, dementia with Lewy bodies and frontotemporal dementia, and increases the likelihood of longevity

**DOI:** 10.1007/s00401-019-02026-8

**Published:** 2019-05-26

**Authors:** Sven J. van der Lee, Olivia J. Conway, Iris Jansen, Minerva M. Carrasquillo, Luca Kleineidam, Erik van den Akker, Isabel Hernández, Kristel R. van Eijk, Najada Stringa, Jason A. Chen, Anna Zettergren, Till F. M. Andlauer, Monica Diez-Fairen, Javier Simon-Sanchez, Alberto Lleó, Henrik Zetterberg, Marianne Nygaard, Cornelis Blauwendraat, Jeanne E. Savage, Jonas Mengel-From, Sonia Moreno-Grau, Michael Wagner, Juan Fortea, Michael J. Keogh, Kaj Blennow, Ingmar Skoog, Manuel A. Friese, Olga Pletnikova, Miren Zulaica, Carmen Lage, Itziar de Rojas, Steffi Riedel-Heller, Ignacio Illán-Gala, Wei Wei, Bernard Jeune, Adelina Orellana, Florian Then Bergh, Xue Wang, Marc Hulsman, Nina Beker, Niccolo Tesi, Christopher M. Morris, Begoña Indakoetxea, Lyduine E. Collij, Martin Scherer, Estrella Morenas-Rodríguez, James W. Ironside, Bart N. M. van Berckel, Daniel Alcolea, Heinz Wiendl, Samantha L. Strickland, Pau Pastor, Eloy Rodríguez Rodríguez, DESGESCO (Dementia Genetics Spanish Consortium), EADB (Alzheimer Disease European DNA biobank), EADB (Alzheimer Disease European DNA biobank), IFGC (International FTD-Genomics Consortium), IPDGC (The International Parkinson Disease Genomics Consortium), IPDGC (The International Parkinson Disease Genomics Consortium), RiMod-FTD (Risk and Modifying factors in Fronto-Temporal Dementia), Netherlands Brain Bank (NBB), Bradley F. Boeve, Ronald C. Petersen, Tanis J. Ferman, Jay A. van Gerpen, Marcel J. T. Reinders, Ryan J. Uitti, Lluís Tárraga, Wolfgang Maier, Oriol Dols-Icardo, Amit Kawalia, Maria Carolina Dalmasso, Mercè Boada, Uwe K. Zettl, Natasja M. van Schoor, Marian Beekman, Mariet Allen, Eliezer Masliah, Adolfo López de Munain, Alexander Pantelyat, Zbigniew K. Wszolek, Owen A. Ross, Dennis W. Dickson, Neill R. Graff-Radford, David Knopman, Rosa Rademakers, Afina W. Lemstra, Yolande A. L. Pijnenburg, Philip Scheltens, Thomas Gasser, Patrick F Chinnery, Bernhard Hemmer, Martijn A. Huisman, Juan Troncoso, Fermin Moreno, Ellen A. Nohr, Thorkild I. A. Sørensen, Peter Heutink, Pascual Sánchez-Juan, Danielle Posthuma, The GIFT (Genetic Investigation in Frontotemporal Dementia and Alzheimer’s Disease) Study Group, G. Coppola, A. M. Karydas, A. Varpetian, T. M. Foroud, A. I. Levey, W. A. Kukull, M. F. Mendez, J. Ringman, H. Chui, C. Cotman, C. DeCarli, B. L. Miller, D. H. Geschwind, Jordi Clarimón, Kaare Christensen, Nilüfer Ertekin-Taner, Sonja W. Scholz, Alfredo Ramirez, Agustín Ruiz, Eline Slagboom, Wiesje M. van der Flier, Henne Holstege

**Affiliations:** 1 Alzheimer Center Amsterdam, Department of Neurology, Amsterdam Neuroscience, Vrije Universiteit Amsterdam, Amsterdam UMC, Amsterdam, The Netherlands; 2 Department of Clinical Genetics, Vrije Universiteit Amsterdam, Amsterdam UMC, Amsterdam, The Netherlands; 3 Department of Neuroscience, Mayo Clinic Florida, Jacksonville, FL 32224 USA; 4 Department of Complex Trait Genetics, Center for Neurogenomics and Cognitive Research, Amsterdam Neuroscience, Vrije Universiteit Amsterdam, Amsterdam UMC, Amsterdam, The Netherlands; 5 Department for Neurodegenerative Diseases and Geriatric Psychiatry, University of Bonn, Bonn, Germany; 6 DZNE, German Center for Neurodegenerative Diseases, Bonn, Germany; 7 Division of Neurogenetics and Molecular Psychiatry, Department of Psychiatry and Psychotherapy, Faculty of Medicine, University Hospital Cologne, Cologne, Germany; 8 Molecular Epidemiology, Leiden University Medical Center, Leiden, The Netherlands; 9 Pattern Recognition and Bioinformatics, Delft University of Technology, Delft, The Netherlands; 10 Research Center and Memory Clinic, Fundació ACE, Institut Català de Neurociències Aplicades, Universitat Internacional de Catalunya, Barcelona, Spain; 11 Centro de Investigacion Biomedica en Red en Enfermedades Neurodegenerativas (CIBERNED), Madrid, Spain; 12 Department of Neurology, Brain Center Rudolf Magnus, University Medical Center Utrecht, Utrecht, The Netherlands; 13 Amsterdam UMC-Vrije Universiteit Amsterdam, Department of Epidemiology and Biostatistics, Amsterdam Public Health Research Institute, Amsterdam, The Netherlands; 14 Interdepartmental Program in Bioinformatics, University of California, Los Angeles, USA; 15 Neuropsychiatric Epidemiology Unit, Department of Psychiatry and Neurochemistry, Institute of Neuroscience and Physiology, Sahlgrenska Academy, Centre for Ageing and Health (AgeCap) at the University of Gothenburg, Gothenburg, Sweden; 16 Max Planck Institute of Psychiatry, Munich, Germany; 17 Department of Neurology, Klinikum rechts der Isar, Technical University of Munich, Munich, Germany; 18 German Competence Network Multiple Sclerosis (KKNMS), Munich, Germany; 19 Movement Disorders and Memory Unit, Department of Neurology, University Hospital Mutua de Terrassa, Barcelona, Spain; 20 Fundacio per la Recerca Biomedica I Social Mutua Terrassa, Terrassa, Barcelona Spain; 21 German Center for Neurodegenerative Diseases (DZNE)-Tübingen, Tübingen, Germany; 22 Hertie Institute for Clinical Brain Research, University of Tübingen, Tübingen, Germany; 23 Memory Unit, Department of Neurology, IIB Sant Pau, Hospital de la Santa Creu i Sant Pau, Universitat Autonoma de Barcelona, Barcelona, Spain; 24 Clinical Neurochemistry Laboratory, Sahlgrenska University Hospital, Mölndal, Sweden; 25 Department of Psychiatry and Neurochemistry, Institute of Neuroscience and Physiology, Sahlgrenska Academy at the University of Gothenburg, Gothenburg, Sweden; 26 Department of Molecular Neuroscience, UCL Institute of Neurology, Queen Square, London, UK; 27 The Danish Aging Research Center, Epidemiology, Biostatistics and Biodemography, Department of Public Health, University of Southern Denmark, Odense, Denmark; 28 Neurodegenerative Diseases Research Unit, National Institute of Neurological Disorders and Stroke, Bethesda, MD 20892-3707 USA; 29 Epidemiology, Biostatistics and Biodemography, Department of Public Health, University of Southern Denmark, Odense, Denmark; 30 Institute of Genetic Medicine, Newcastle University, Newcastle upon Tyne, NE1 3BZ UK; 31 Department of Clinical Neurosciences, University of Cambridge, Cambridge, CB2 0QQ UK; 32 Institut für Neuroimmunologie und Multiple Sklerose (INIMS), Universitätsklinikum Hamburg‐Eppendorf, Hamburg, Germany; 33 Department of Pathology (Neuropathology), Johns Hopkins University Medical Center, Baltimore, MD USA; 34 Instituto Biodonostia, San Sebastian, Spain; 35 University Hospital “Marques de Valdecilla”, Santander, Spain; 36 IDIVAL, Santander, Spain; 37 Institute of Social Medicine, Occupational Health and Public Health (ISAP), University of Leipzig, Leipzig, Germany; 38 Department of Neurology, University of Leipzig, Leipzig, Germany; 39 Newcastle Brain Tissue Resource, Edwardson Building, Institute of Neuroscience, Newcastle University, Newcastle upon Tyne, NE4 5PL UK; 40 Cognitive Disorders Unit, Department of Neurology, Hospital Universitario San Sebastian, San Sebastian, Spain; 41 Department of Radiology and Nuclear Medicine, Amsterdam Neuroscience, Vrije Universiteit Amsterdam, Amsterdam UMC, Amsterdam, The Netherlands; 42 Department of Primary Medical Care, Center for Psychosocial Medicine, University Medical Center, Hamburg-Eppendorf, Germany; 43 Centre for Clinical Brain Sciences, University of Edinburgh, Edinburgh, EH4 2XU UK; 44 Department of Neurology, Klinik für Neurologie mit Institut für Translationale Neurologie, University of Münster, Münster, Germany; 45 Department of Neurology, Mayo Clinic Minnesota, Rochester, MN 55905 USA; 46 Department of Psychiatry and Psychology, Mayo Clinic Florida, Jacksonville, FL 32224 USA; 47 Department of Neurology, Mayo Clinic Florida, Jacksonville, FL 32224 USA; 48 Fundación Instituto Leloir-IIBBA-CONICET, Buenos Aires, Argentina; 49 Department of Neurology, University of Rostock, Rostock, Germany; 50 Laboratory of Neurogenetics, National Institute on Aging, National Institutes of Health, Bethesda, MD USA; 51 Department of Neurology, Hospital Universitario San Sebastian, San Sebastian, Spain; 52 Department of Neurology, Johns Hopkins University Medical Center, Baltimore, MD 21287 USA; 53 Center of Neurology, Department of Neurodegenerative diseases, Hertie-Institute for Clinical Brain Research, University of Tuebingen, Tuebingen, Germany; 54 MRC Mitochondrial Biology Unit, University of Cambridge, Cambridge, CB2 0QQ UK; 55 Munich Cluster for Systems Neurology (SyNergy), Munich, Germany; 56 Department of Sociology, VU University, Amsterdam, The Netherlands; 57 Research Unit of Gynecology and Obstetrics, Department of Clinical Research, University of Southern Denmark, Odense, Denmark; 58 Novo Nordisk Foundation Center for Basic Metabolic Research, Section of Metabolic Genetics, Copenhagen, Denmark; 59 Department of Public Health, Section of Epidemiology, Faculty of Health and Medical Sciences, University of Copenhagen, Copenhagen, Denmark; 60 MRC Integrative Epidemiology Unit, Bristol University, Bristol, UK; 61 Clinical Biochemistry and Pharmacology, Odense University Hospital, Odense, Denmark; 62 Department of Clinical Genetics, Odense University Hospital, Odense, Denmark; 63 Dutch Society for Research on Ageing, Leiden, The Netherlands; 64 Delft Bioinformatics Lab, Delft University of Technology, Delft, The Netherlands

**Keywords:** Alzheimer’s disease, Frontotemporal dementia, Dementia with Lewy bodies, Progressive supranuclear palsy, Parkinson’s disease, Amyotrophic lateral sclerosis, Multiple sclerosis, Neurodegenerative disease, Longevity, PLCG2, Phospholipase C Gamma 2

## Abstract

The genetic variant rs72824905-G (minor allele) in the *PLCG2* gene was previously associated with a reduced Alzheimer’s disease risk (AD). The role of *PLCG2* in immune system signaling suggests it may also protect against other neurodegenerative diseases and possibly associates with longevity. We studied the effect of the rs72824905-G on seven neurodegenerative diseases and longevity, using 53,627 patients, 3,516 long-lived individuals and 149,290 study-matched controls. We replicated the association of rs72824905-G with reduced AD risk and we found an association with reduced risk of dementia with Lewy bodies (DLB) and frontotemporal dementia (FTD). We did not find evidence for an effect on Parkinson’s disease (PD), amyotrophic lateral sclerosis (ALS) and multiple sclerosis (MS) risks, despite adequate sample sizes. Conversely, the rs72824905-G allele was associated with *increased* likelihood of longevity. By-proxy analyses in the UK Biobank supported the associations with both dementia and longevity. Concluding, rs72824905-G has a protective effect against multiple neurodegenerative diseases indicating shared aspects of disease etiology. Our findings merit studying the PLCγ2 pathway as drug-target.

**Electronic supplementary material:**

The online version of this article (10.1007/s00401-019-02026-8) contains supplementary material, which is available to authorized users.

## Introduction

The protein product of the phospholipase Cγ2 (*PLCG2*) gene is involved in the transmembrane transduction of immune signals [30, 42, 45] that determine the fate and function of various immune cell types, both in the periphery and the brain [42, 45]. It is known that gain-of-function mutations in the *PLCG2* gene cause autoimmune disorders [40, 46, 58, 59] and resistance to treatment of chronic lymphocytic leukemia [56].

In 2017, a genome-wide association (GWA) study of Alzheimer’s disease (AD) showed that the rare nonsynonymous variant in the *PLCG2* gene (rs72824905-G; p.Pro522Arg; NC_000016.9:g.81942028C > G) reduced AD risk (OR = 0.68, *p* = 5.4 × 10^−10^) [47]. In both mouse and human brain tissues, *PLCG2* has been shown to be overexpressed > 6-log fold in microglia compared to other brain cells [12]. Further, *PLCG2* has higher expression levels in pathologically affected brain regions of AD patients, which seems to be driven by microgliosis [7]. Since microglia are the brain’s immune cells, these findings suggest an important role for *PLCG2* in the neural immune response. Next to *PLCG2,* GWA studies of AD identified additional immune- and microglia-related genes that associate with AD, e.g. the triggering receptor expressed on myeloid cells 2 (*TREM2*) gene and pathway analysis based on these same GWA studies indicate that the immune system plays a key role in the development of AD [47]. Likewise, human genetic studies imply the immune system plays a role in other neurodegenerative diseases such as frontotemporal dementia (FTD) [3], Parkinson’s disease (PD) [13], and multiple sclerosis (MS) [17, 20, 41]. We reasoned that next to AD, *PLCG2*-related immune signaling may be involved in the etiology of these other neurodegenerative diseases. This led us to question whether the rs72824905-G variant in *PLCG2* is also associated with a reduced risk of other neurodegenerative diseases.

Here, we tested whether rs72824905-G protects against other neurodegenerative diseases. We first tested whether rs72824905-G associates with reduced risk of AD, FTD, dementia with Lewy bodies (DLB), progressive supranuclear palsy (PSP), PD, amyotrophic lateral sclerosis (ALS) and MS. Since a reduced risk of neurodegenerative diseases could lead to an increased likelihood to survive to old age, we tested whether rs72824905-G associated with longevity.

## Materials and methods

### Study populations and genotyping

We present a short description of 16 cohorts, often including multiple sites or studies, which contributed to this manuscript in Suppl. Table 1, Online Resource. Studies were approved by corresponding ethics committees and informed consent was obtained for all participants (Suppl. Table 1, Online Resource). Study characteristics (age, percentage female, apolipoprotein E (APOE) status and age) are described in Suppl. Table 2, Online Resource. In most cohorts, the average age of the controls was lower than that of cases (Suppl. Fig 3, Online Resource). We determined rs72824905-G genotypes (NC_000016.9:g.81942028C > G, p.Pro522Arg) using direct genotyping with a variety of genotyping arrays or TaqMan genotyping. If direct genotyping was not available, we used imputation to 1000 Genomes phase I version 3 [15] or the Haplotype Reference Consortium (HRC) reference panels [37]. Details on genotyping or imputation by study can be found in Suppl. Table 3, Online Resource. We studied participants from European descent.

### Study populations of AD, FTD, DLB and PSP patients

We compared rs72824905-G genotypes in a total of 4,985 AD patients and 9,238 controls from eight cohorts. All samples were independent from Sims et al. [47], but include the samples from Conway et al. [7]. We compared in total 2,437 FTD patients with 10,647 controls from four studies and two consortia. Further, we studied 1446 DLB patients with 5509 controls from five cohorts and 882 PSP patients with 3187 controls from five cohorts. Details on sample size by cohort and which cohort contributed to which analysis can be found in Suppl. Table 2, Online Resource.

### Study populations of ALS, PD and MS patients

To study the association of rs72824905-G with ALS, PD and MS, we obtained summary statistics from existing GWAS meta-analyses, see Suppl. Table 1, 2, 3, Online Resource, for study descriptions. We present results of a combined total of 28,448 PD patients that were compared with 108,438 controls: data from 27,595 PD patients and 106,951 controls from the International Parkinson Disease Genomics Consortium (IPDGC) [39] were combined with data from 853 PD patients and 1,487 controls from the Mayo Clinic. Furthermore, we studied 10,953 ALS patients and 20,673 controls, which represents the subset of the data presented by van Rheenen et al. [54], for which rs72824905-G was imputed with sufficient quality (imputation quality > 0.3). Last, we studied 4476 MS patients and 5714 controls which were previously described by Dankowski et al. [8].

### Study populations of longevity

We investigated the association of rs72824905-G with longevity in five different cohorts; in total, we compared 3516 individuals who reached at least 90 years with 9677 control individuals who died before age 90 years or were last screened before 90 years (Suppl. Table 1–3, Online Resource). The data from Tesi et al. [51] were included in this study. A subset of 1136 Dutch long-lived individuals for whom follow-up data until death were available [22] was included. In this subset, we compared the survival of carriers of rs72824905-G with non-carriers.

### Studies of dementia and longevity by-proxy in the UK Biobank

The UK Biobank is a study of genetic and health of a half million people from the United Kingdom [49]. Information from parents or first-degree relatives can be used as a proxy-phenotype for the participants [34]. In this study, we used maternal and paternal history of Alzheimer’s/dementia as proxy for dementia [34, 36] and the reported age of the parents (at completing the survey or death) as proxy phenotype for longevity [44]. In the UK Biobank, the rs72824905-G variant was imputed using the available genotyping arrays and the HRC-reference panel as previously described [25]. The maternal and paternal by-proxy phenotypes were analyzed separate using the genotypes of the participants and the results were meta-analyzed.

We compared rs72824905-G genotypes of 32,262 participants whose mother was reported to have dementia with the genotypes of 346,999 participants whose mothers did not have dementia. Likewise, we compared 16,968 participants whose father had dementia with 358,468 whose fathers did not have dementia.

For the analysis of longevity-by-proxy, we chose the age of 90 years as a cut-off for the minimum age reached by the parents. By principle, phenotype by-proxy analyses suffer from dilution effect [34, 36]; therefore, a more extreme parental age cut-off of 95 years was also studied. In this analysis, we compared 35,256 UK Biobank participants who had a mother who reached at least 90 years (7790 mothers reached the age of 95 years) with 342,810 participants whose mother did not reach 90 years of age. Likewise, we compared 17,558 UK Biobank participants with a father who reached at least 90 years (3,043 fathers reached the age of 95 years) with 353,100 participants whose father did not reach 90 years of age.

### Statistical analysis

R (version 3.5.1) was used for all analysis [50]. Logistic regression models were fitted within studies to assess the association of rs72824905-G with AD, FTD, DLB, and PSP patients, and long-lived individuals, compared to controls. For each study, we calculated the odds ratio’s (OR) and 95% confidence intervals (CI). We accounted for population substructure by adjusting for principal components or by comparing cases and controls from the same study or country of origin. We meta-analyzed the effect estimates (log(OR)) from the studies using inverse-variance fixed-effect meta-analyses (R-package ‘rmeta’ v3.0). The fraction of variance that is due to heterogeneity was estimated by the I^2^ statistic [21]. We visualized survival of rs72824905-G carriers compared to non-carriers using Kaplan–Meier curves. Differences in survival were tested using a Cox proportional hazards model correcting for (age at inclusion, sex and relatedness).

For MS, the results originate from a single study, which used ancestry principal components (PCs) to adjust for population stratification [8]. The statistical methods of the GWAS meta-analyses of ALS and PD were previously described [39, 54]. In short, individual cohorts calculated logistic regression models and then summary statistics of cohorts were combined using inverse-variance fixed-effect meta-analyses. PCs were used to adjust for population stratification. Analysis in the UK Biobank were performed using logistic regression models adjusted for genotyping array and the first 12 PCs. Effect estimates of the paternal and maternal analysis were combined using inverse-variance fixed-effect meta-analysis (R-package ‘rmeta’ v3.0). We reported two-sided *p* values and considered *p* values < 0.05 as significant; *p* values are not corrected for multiple testing.

### Power analysis

For all diseases studied, we performed power analysis using the online tool Genetic Association Study (GAS) power Calculator implementing the methods described in Skol et al. [48]. We calculated power of our analysis to attain a *p* value of 0.05 and used the total number of cases and controls from our analysis. We assumed an additive model, a minor allele frequency of 0.009 and a disease frequency of 0.01 for all diseases (higher disease frequency assumption would lead to higher power estimates). We report the power for an OR between 1 and 2. This corresponds to protective OR (the inverse OR = 1/OR) between 0.50 and 1.

## Results

An overview of study sample, contributing studies, corrections applied by study and counts of carriers split by case–control status is shown in Table 1.

**Table 1:** Study sample description

Trait	Consortium or combined cohort name	Studies/sites included	Corrections	Cases			Controls		
				*N*	N-carriers	MAF	*N*	N-carriers	MAF
AD	Amsterdam UMC	ADC, NBB, LASA	PC1-3	1893	24	0.63	2571	64	1.24
	Brain compendium	Keogh et al. [29]	None	277	0	0	362	6	0.83
	Mayo Clinic	Conway et al. [7]	None	1477	19	0.64	1487	29	0.98
	NDRU cohort	NDRU cohort	None	527	7	0.66	343	8	1.17
	Spanish cohorts	Valdecilla Cohort, Fundació ACE, Oviedo, Sant Pau (SPIN cohort), San Sebastian	None	23	0	0	746	10	0.67
	Swedish studies	GBC Studies, Clinical AD cohort sweden.	None	564	6	0.53	3480	61	0.88
	UCLA/UCSF GIFT	Chen et al. [6]	None	224	0	0	249	10	2.01
	Combined AD			4985	56	0.56	9238	188	1.02
DLB	Amsterdam UMC	ADC, NBB, LASA	PC1-3	189	2	0.53	2571	64	1.24
	Brain compendium	Keogh et al. [29]	None	97	1	0.52	362	6	0.83
	Mayo Clinic	Conway et al. [7]	None	306	2	0.33	1487	29	0.98
	NDRU cohort	NDRU cohort	None	622	8	0.64	343	8	1.17
	Spanish cohorts	Valdecilla Cohort, Fundació ACE, Oviedo, Sant Pau (SPIN cohort), San Sebastian	None	232	3	0.65	746	10	0.67
	Combined DLB			1446	16	0.55	5509	117	1.06
FTD	Amsterdam UMC	ADC, NBB, LASA	PC1-3	231	1	0.22	2571	64	1.24
	Brain compendium	Keogh et al. [29]	None	93	2	1.08	362	6	0.83
	IFGC	Ferrari et al. [11]	None	1360	22	0.81	5059	118	1.17
	RiMoD-FTD	(Consortium)	None	255	3	0.59	1660	38	1.17
	Spanish cohorts	Valdecilla Cohort, Fundació ACE, Oviedo, Sant Pau (SPIN cohort), San Sebastian	None	366	1	0.14	746	10	0.67
	UCLA/UCSF GIFT	Chen et al. [6]	None	132	2	0.76	249	10	2.01
	Combined FTD			2437	31	0.64	10,647	246	1.19
PSP	Brain compendium	Keogh et al. [29]	None	17	1	2.94	362	6	0.83
	Mayo Clinic	Conway et al. [7]	None	231	9	1.95	1487	29	0.98
	NDRU cohort	NDRU cohort	None	613	11	0.9	343	8	1.17
	UCLA/UCSF GIFT	Chen et al. [6]	None	12	0	0	249	10	2.01
	Combined PSP			873	21	1.20	2441	53	1.09
PD	IPDGC	Nalls et al. [39]	≥3PCs	27,595	340^a^	0.81^a^	106,951	391^a^	0.81^a^
	Mayo Clinic	Conway et al. [7]	None	853	18	1.06	1487	29	0.98
MS	KKNMS	Dankowski et al. [3]	PC1 and 2	4476	82	0.92	5714	107	0.94
ALS	Project MinE	Van Rheenen et al. [8]	PC1-4	10,953	214	0.98	20,673	385	0.93
longevity	AgeCoDe	AgeCoDe	None	462	14	1.52	861	19	1.12
	Amsterdam UMC	100-Plus Study, LASA, NBB	PC1-3	293	16	2.73	2571	64	1.24
	Danish studies	Multiple Danish studies	None	853	10	0.59	2793	33	0.59
	Leiden Longevity Study	LLS, GEHA-NL	None	1138	28	1.23	743	11	0.74
	GBC Studies	GBC Studies	None	770	16	1.04	2709	45	0.83
	Combined longevity			3516	84	1.19	9677	172	0.89

Consortium or combined cohort name corresponds to the name used in the figures of this manuscript. Studies/sites included or reference to cohort shows the studies combined to form one site (if more then one). Additional information on studies included can be found in supplementary Table 2. If studies/sites include a reference, the exact methods described in the reference were used to obtain the genotypes and association results

*AD* Alzheimer’s disease, *FTD* frontotemporal dementia, *DLB* dementia with Lewy bodies, *PSP* progressive supranuclear palsy, *PD* Parkinson’s Disease, *ALS* Amyotrophic Lateral Sclerosis, *MS* multiple sclerosis, *MAF* Minor allele frequency, *ADC* Amsterdam Dementia Cohort, *NBB* Netherlands Brain Bank, *LASA* Longitudinal Aging Study Amsterdam, *GEHA* Genetics of Healthy Ageing Study, NL, *AgeCoDe* German Study on Ageing, Cognition and Dementia in Primary Care Patients, *GBC* Gothenburg Birth Cohort Studies, *IFGC* International FTD-Genomics Consortium, *IPDGC* The International Parkinson Disease Genomics Consortium, *KKNMS* German Competence Network Multiple Sclerosis, *LLS* Leiden Longevity study, *RiMoD-FTD* Risk and modifying factors in Frontotemporal Dementia, *UCLA/UCSF* Genetic Investigation in Frontotemporal Dementia and Alzheimer’s Disease (GIFT) Study

^a^The number of carriers and minor allele frequency were calculated in a subset of 21092 cases and 23896 controls. No combined estimate of MAF can be given

### Association with brain diseases

**Fig. 1:**
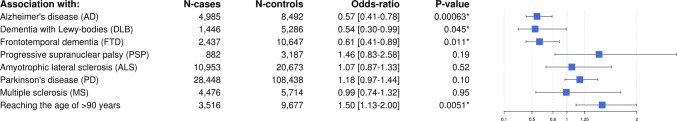
Association results of rs72824905-G with seven brain diseases and longevity. *P values < 0.05. Numbers (*N*) of cases (patients or long-lived individuals) and controls studied. The figure shows the odds-ratio (box) of the rs72824905-G with the 95% confidence intervals (whiskers)

We replicated the association of rs72824905-G in *PLCG2* with a reduced AD risk (OR = 0.57, *p *= 6.0 × 10^−4^, *I*^2^ = 0%). In addition, we found that rs72824905-G associated with a reduced risk of both DLB (OR = 0.54, *p *= 0.045, *I*^2^ = 0%) and FTD (OR = 0.61, *p* = 0.011, *I*^2^ = 0%). In contrast, we found no evidence that rs72824905-G is associated with PSP (OR = 1.46, *p *= 0.19, *I*^2^ = 0%), ALS (OR = 1.07, *p *= 0.52, *I*^2^ = 0%), PD (OR = 1.18, *p *= 0.10, *I*^2^ = 0) and MS (OR = 0.99, *p *= 0.95). The association of rs72824905-G with these seven brain diseases is shown in Fig. 1. In Suppl. Figs. 2–7, Online Resource, we show the association estimates for each study separately in the meta-analyses for AD, DLB, FTD, PSP, ALS and PD (the MS study consisted of a single study).

### Association with longevity

In line with a reduced risk of neurodegenerative diseases, we found that rs72824905-G associated with a 1.49 (95% CI 1.12–1.98) increased likelihood (*p* = 6.3 × 10^−3^, *I*^2^ = 0%) to reach the age of 90 years. Although no heterogeneity was observed between studies, it is of interest that a cohort of centenarians who were selected based on being 100 years old and cognitively healthy (description of ‘100-plus Study’ in Suppl. Table 1, Online Resource) was most enriched with rs72824905-G (OR = 2.36, 95% CI 1.34–4.15, *p* = 2.8 × 10^−3^) (Suppl. Fig 8, Online Resource). Next, we tested whether carrying the rs72824905-G variant was associated with longer survival after the age of 90 years in 1,136 Dutch long-lived individuals of which 96.3% were followed until death [median age at inclusion 93.2, IQR 91.6–95.0 years, 63% female; mean survival after inclusion was 3.3 years; inter quartile range (IQR) 1.4–5.8 years]. We found that 28 carriers survived a median of 4.7 years (IQR = 1.9–7.4) while 1108 non-carriers survived a median of 3.3 years (IQR = 1.4–5.8) (Suppl. Fig 9, Online Resource). However, the difference was not significant (HR 0.75, 95% CI 0.51–1.09, *p *= 0.078), likely due to the low number of rs72824905-G carriers in the analysis, as a consequence of variant rareness (MAF ~ 1%).

### Association with by-proxy dementia and longevity

**Fig. 2:**
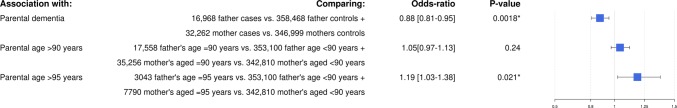
Association results of rs72824905-G with dementia by-proxy and longevity by-proxy analysis in the UK Biobank. *P values < 0.05. The figure shows the odds-ratio (box) of the rs72824905-G with the 95% confidence intervals (whiskers)

In line with the protection against AD, the by-proxy analysis showed that *PLCG2* variant carriers had a reduced risk of having a parent with dementia, OR = 0.88 (0.81–0.95, *p *= 1.9 × 10^−3^) (Fig. 2). Next, we tested the association of rs72824905-G with longevity-by-proxy. Carriers of the rs72824905-G variant did not have an increased likelihood of having a parent who reached 90 years of age compared to non-carriers (OR = 1.05, *p* = 0.24). However, carriers did have an increased likelihood of having a parent who reached 95 years (OR = 1.19, *p* = 2.1 × 10^−2^). The threshold of 95 years was chosen as too few parents reached the age of 100 years.

### Power analysis

Power analysis (Suppl. Fig 10, Online Resource) showed that the PD, MS and ALS analysis had adequate statistical power (power > 0.8) to detect a protective association (*p* = 0.05) with an OR ~ 0.68 (the OR for AD reported in Sims et al. [47].). The PSP analysis had the lowest statistical power (0.32 at the expected OR = 0.67).

## Discussion

A recent study showed the protective effect against AD risk of the p.Pro552Arg nonsynonymous amino acid change in *PLCG2* (rs72824905-G) [47]. We replicated this protective effect in independent AD patients and controls. Additionally, we found that the variant also protected against FTD and DLB, but *not* against ALS, PD and MS. The analysis of PSP was inconclusive because of insufficient power. We also found that rs72824905-G associated with increased likelihood of longevity, which is according to expectations, since overall dementia is the leading cause of death at older age [1]. Indeed, the strongest effect of *PLCG2* variant was observed in cognitively healthy centenarians, individuals where an absence of dementia and extreme longevity is combined. Our findings were supported by analyses of by-proxy phenotypes for dementia and longevity in the UK Biobank. Taken together, the association of the rs72824905-G variant with a decreased risk of multiple dementia types and the increased risk of longevity warrants thorough investigation of the molecular mechanisms underlying this protective effect.

**Fig. 3:**
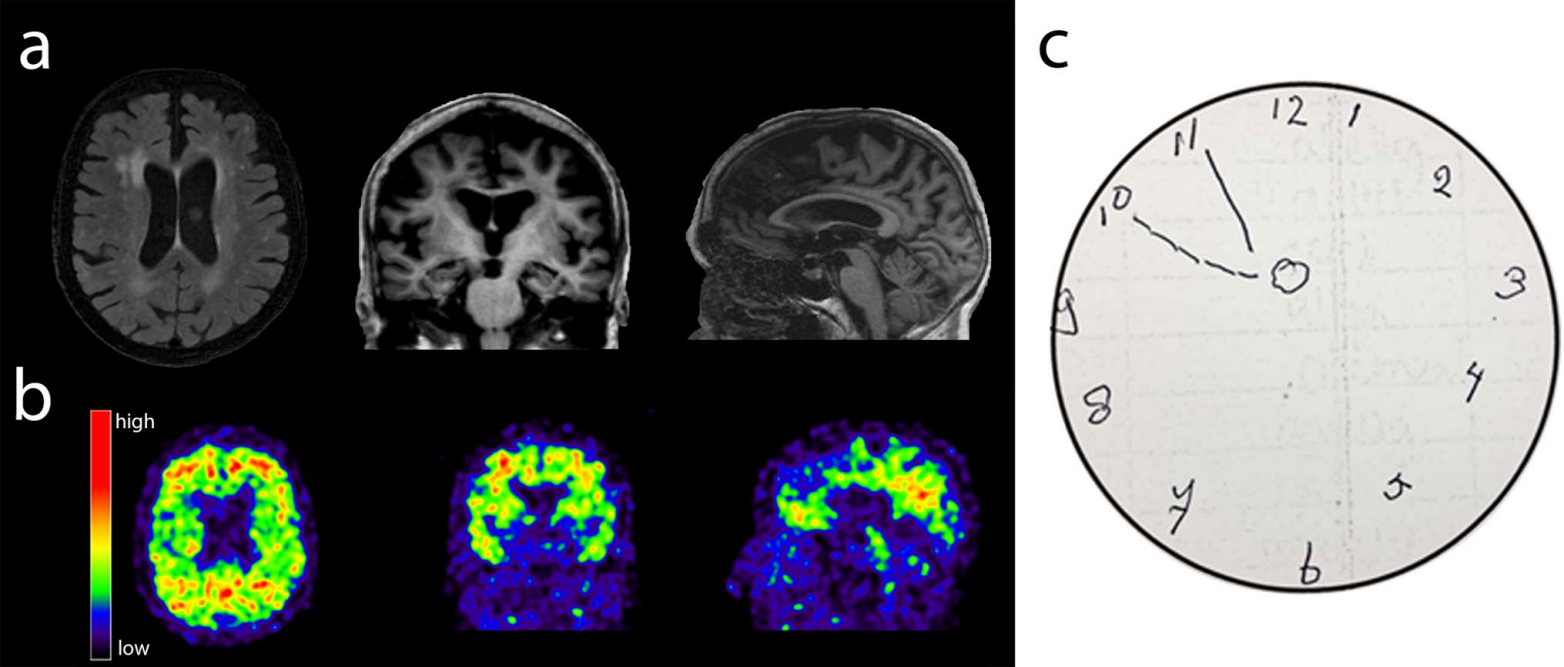
MRI scan and PiB-PET scan, of a 102-year-old centenarian carrying the homozygote *APOE* ɛ4 genotype as well as the rs72824905-G allele in *PLCG2*. MRI scan (Titan 3T MR scanner) shows some hippocampal atrophy (MTA grade 2), some global cortical atrophy (GCA-scale grade 1), but pronounced posterior cortical atrophy (grade 2), moderate white matter hyperintensities (Fazekas grade 2), no lacunar infarcts or microbleeds. PET-PiB (scan after admission of 396 MBq C-11 PIB, 20-min image starting 90 min after administration): Abnormal retention in the posterior cingulate/precuneus and frontal lobes. Neuropsychological testing around time of scanning showed average performance on global cognitive functioning/MMSE, memory, attention, working memory, fluency and visuo-spatial tests compared to the cohort of cognitively healthy centenarians. The result of the clock drawing test is shown. The patient was asked to draw a clock and put the time at 10 before 11

Thus far, the common *APOE* ɛ4 allele and the rare *TREM2*.R47H variant are strong genetic risk factors shared across AD, FTD and DLB (not *TREM2* [55]). [2, 11, 18, 27, 47] The *HLA*-locus and the microtubule-associated protein tau (*MAPT*) loci (not individual variants) also have (suggestive) effects on the risks of AD, FTD and DLB [3, 11, 18, 43]. The *APOE* gene has been implied in a multitude of pathways [52], *TREM2* and *HLA* are involved in immunity [2], and *MAPT* encodes the tau protein. These shared genetic risk factors indicate a partial overlap in AD, DLB and FTD etiology. It is of interest that, like the *PLCG2* variant, *APOE* and the *HLA*-*DR* locus were also associated with longevity [4, 10, 28, 38]. A possible explanation is that *APOE, PLCG2* and *HLA* are involved in the processing of accumulated aging-associated proteins [9]. In line with this hypothesis, rs72824905-G is associated with reduced pTau_181_ in the CSF of memory clinic patients with pathologic Aβ_1-42_ CSF levels (L. Kleineidam et al. submitted). It is well known that having a dementia-associated neurodegenerative disease is associated with shorter life-span [57]. Conversely, resilience to diseases is associated with a longer life-span [24]. It is likely that the association of rs72824905-G with longevity is due to the protection against dementia-associated neurodegenerative diseases. However, with the available data we cannot exclude that rs72824905-G has an independent effect of rs72824905-G on the risk of longevity and/or the risk of maintaining cognitive health. In line with this observation, we anecdote one cognitively healthy centenarian who is homozygous for the *APOE* ɛ4 risk allele, but also carried the rs72824905-G allele. On MRI scan and amyloid scan (PiB-PET), this person has some global atrophy and only amyloid-β positivity in the precuneus and in the frontal lobes (Fig. 3). At the age of 90 years, the dementia risk for homozygous carriers of the *APOE* ɛ4 genotype is approximately 80% [53] and virtually all are amyloid positive by age 90 [26]. The literature reports only a handful of centenarians who are homozygous for the *APOE* ɛ4 allele [14, 16, 23]. It is unknown if these individuals were cognitively healthy. This case shows that cognitively healthy aging in the presence of the *APOE* ɛ4ɛ4 genotype is possible, likely due to the protective effect of other genetic variants, such as the rs72824905-G variant in *PLCG2* [26, 53].

The mechanism that explains the protective effect of rs72824905-G variant in the *PLCG2* gene is currently unclear. We find that the associations of the rs72824905-G variant with disease risk differ between diseases that have overlapping pathological features. For example, we observe that carrying the rs72824905-G variant is protective against DLB, but not against PD, while a common characteristic for both diseases is the presence of α-synuclein-positive Lewy bodies. The same holds for pathologies associated with the FTD-ALS and PSP spectrum of diseases (e.g. TDP-43, FUS inclusions as well as aggregations of tau). The observation that our results do not point to a single pathological condition does not preclude that *PLCG2* is involved in a single biological process. In fact, determining the involvement of the *PLCG2*-related pathway might be an asset in pathological classifications of diseases, e.g. differentiating between DLB and PD. Thus far, only one publication investigated the functional effect of the rs72824905-G variant in in vitro experiments [35]. The authors suggest that in the mouse and human brain, PLCɣ2 is expressed in microglia [12]. They show that PLCɣ2 mRNA co-localized with microglia-specific markers in healthy brain tissue and is expressed in microglia near amyloid-β plaques in an APP mouse model [35]. Furthermore, functional characterization of PLCɣ2 with the p.Pro552Arg amino acid substitution suggested only a slight increase in activity compared to wild-type PLCɣ2 [35]. While additional functional experiments will be needed to confirm these findings, these experiments suggest that the functional changes induced by the PLCɣ2 p.Pro552Arg genetic variant may be subtle and, therefore, difficult to pinpoint. This is according to expectations, as major changes to the immune system will most likely be harmful. Indeed, known germline mutations in *PLCG2* cause the immune disorders PLAID (*PLCG2*-associated antibody deficiency and immune dysregulation) and APLAID (autoinflammatory PLAID) [40, 46, 59] while somatic variants in PLCɣ2 are associated with resistance to treatment of leukemia [56] (reviewed in Koss et al. [32].). The mutations that cause PLAID and APLAID contribute to a strong hyperactivation of PLCɣ2 upon activation. In the case of APLAID (caused by a p.Ser707Tyr substitution), the auto-inflammation has been suggested to be partially driven by PLCɣ2-dependent activation of the pyrin (PYD)-domain-containing protein 3 (*NLRP3*) inflammasome [5]. The potential of *PLCG2* to activate the inflammasome is further supported by in vitro experiments [31]. The *NLRP3* inflammasome is a crucial signaling node in microglia that ultimately controls the maturation of pro-inflammatory interleukin (IL)-1β and IL-18 [19] and has been linked to a multitude of neurodegenerative diseases [60]. Although functional studies will need to elucidate the effects of the rs72824905-G on PLCɣ2 function, we speculate that subtle changes in the NLRP3 inflammasome activation may explain its protective effect.

### Strengths and weaknesses

The most important strength of our study is that we investigated the effect of the rs72824905-G variant in seven neurological diseases in more than 53,000 patients and almost 150,000 controls. The AD cases and controls studied here were all independent from the AD patients and controls in which the protective effect of rs72824905-G was first identified [47], but includes the samples used in Conway et al. [7] and Tesi et al. [51]. This report offers a robust replication of the protection against AD. Some may argue that the protective effect observed in FTD and DLB cases is driven by misclassified AD cases. However, the effect size of rs72824905-G in these cases is very similar to the protective effect in AD, which makes it unlikely that the effect can be ascribed purely to misclassified AD. Moreover, the age of the controls was mostly younger than that of cases, making the protection from dementia not a longevity effect.

The large numbers under study were necessary because rs72824905-G has a minor allele frequency (MAF) ~ 1% in European ancestry populations, which makes it a relatively rare genetic variant. Therefore, we ensured that our samples provided adequate statistical power to observe a similar protective effect of rs72824905-G against other neurodegenerative diseases. Despite the large sample sizes, we found no evidence for this effect in our sample of PD, ALS and MS, which makes it unlikely that larger meta-analyses will observe an association between rs72824905-G and these three diseases. An association of rs72824905-G with an increased risk of PSP has been reported previously [7]. In our analysis, which includes additional PSP cases, we were not able to replicate this finding. Larger studies are needed to determine the association with PSP. Including as large as possible samples came with the consequence that we were not able to correct for population stratification using ancestry principal components in all studies. Therefore, we used PCs were possible and further matching cases and controls by study or country of origin. Finally, we indicate that the identified effects need to be replicated in other ethnicities in which rs72824905-G occurs. We note that in some ethnicities rs72824905-G plays no role as the frequency is very low in African (MAF = 0.0012) and African-American populations (MAF = 0.0004), and is not observed in East Asian [7, 33].

## Conclusions

Our study shows that the rs72824905-G allele in *PLCG2* associates with a decreased risk for AD, FTD, DLB and concurrently with an increased chance of longevity. The protective effect of the rs72824905-G allele was not observed in ALS, PD and MS cases, which suggests that PLCG2-associated processes overlap in the etiology of AD, FTD and DLB, but not in the etiologies of ALS, PD and MS (PSP too little power). Explaining the protective effect of the PLCγ2 protein on brain immune function may contribute to the design of successful therapeutic intervention strategies applicable to those at risk for neurodegenerative diseases.

## Electronic supplementary material

Below is the link to the electronic supplementary material.

Supplementary material 1 (XLSX 77 kb)

Supplementary material 2 (PDF 972 kb)
