# Correction to: A nonsynonymous mutation in *PLCG2* reduces the risk of Alzheimer’s disease, dementia with Lewy bodies and frontotemporal dementia, and increases the likelihood of longevity

**DOI:** 10.1007/s00401-019-02107-8

**Published:** 2020-01-18

**Authors:** Sven J. van der Lee, Olivia J. Conway, Iris Jansen, Minerva M. Carrasquillo, Luca Kleineidam, Erik van den Akker, Isabel Hernández, Kristel R. van Eijk, Najada Stringa, Jason A. Chen, Anna Zettergren, Till F. M. Andlauer, Monica Diez-Fairen, Javier Simon-Sanchez, Alberto Lleó, Henrik Zetterberg, Marianne Nygaard, Cornelis Blauwendraat, Jeanne E. Savage, Jonas Mengel-From, Sonia Moreno-Grau, Michael Wagner, Juan Fortea, Michael J. Keogh, Kaj Blennow, Ingmar Skoog, Manuel A. Friese, Olga Pletnikova, Miren Zulaica, Carmen Lage, Itziar de Rojas, Steffi Riedel-Heller, Ignacio Illán-Gala, Wei Wei, Bernard Jeune, Adelina Orellana, Florian Then Bergh, Xue Wang, Marc Hulsman, Nina Beker, Niccolo Tesi, Christopher M. Morris, Begoña Indakoetxea, Lyduine E. Collij, Martin Scherer, Estrella Morenas-Rodríguez, James W. Ironside, Bart N. M. van Berckel, Daniel Alcolea, Heinz Wiendl, Samantha L. Strickland, Pau Pastor, Eloy Rodríguez Rodríguez, DESGESCO (Dementia Genetics Spanish Consortium), EADB (Alzheimer Disease European DNA biobank), IFGC (International FTD-Genomics Consortium), IPDGC (The International Parkinson Disease Genomics Consortium), S. Mead, M. Synofzik, J. C. van Swieten, I. Leber, R. Ferrari, D. G. Hernandez, M. A. Nalls, J. D. Rohrer, A. Ramasamy, J. B. J. Kwok, C. Dobson-Stone, P. R. Schofield, G. M. Halliday, J. R. Hodges, O. Piguet, L. Bartley, E. Thompson, B. Borroni, A. Padovani, C. Cruchaga, N. J. Cairns, L. Benussi, G. Binetti, R. Ghidoni, G. Forloni, D. Albani, D. Galimberti, C. Fenoglio, M. Serpente, E. Scarpini, R. Blesa, M. Landqvist Waldö, K. Nilsson, C. Nilsson, I. R. A. Mackenzie, G.-Y. R. Hsiung, D. M. A. Mann, J. Grafman, C. M. Morris, J. Attems, T. D. Griffiths, I. G. McKeith, A. J. Thomas, P. Pietrini, E. D. Huey, E. M. Wassermann, A. Baborie, E. Jaros, M. C. Tierney, C. Razquin, S. Ortega-Cubero, E. Alonso, R. Perneczky, J. Diehl-Schmid, P. Alexopoulos, A. Kurz, I. Rainero, E. Rubino, L. Pinessi, E. Rogaeva, P. St George-Hyslop, G. Rossi, F. Tagliavini, G. Giaccone, J. B. Rowe, J. C. M. Schlachetzki, J. Uphill, J. Collinge, A. Danek, V. M. Van Deerlin, M. Grossman, J. Q. Trojanowski, J. van der Zee, C. Van Broeckhoven, S. F. Cappa, D. Hannequin, V. Golfier, M. Vercelletto, A. Brice, B. Nacmias, S. Sorbi, S. Bagnoli, I. Piaceri, J. E. Nielsen, L. E. Hjermind, M. Riemenschneider, M. Mayhaus, B. Ibach, G. Gasparoni, S. Pichler, W. Gu, M. N. Rossor, N. C. Fox, J. D. Warren, M. G. Spillantini, H. R. Morris, P. Rizzu, J. S. Snowden, S. Rollinson, A. Richardson, A. Gerhard, A. C. Bruni, R. Maletta, F. Frangipane, C. Cupidi, L. Bernardi, M. Anfossi, M. Gallo, M. E. Conidi, N. Smirne, M. Baker, K. A. Josephs, J. E. Parisi, W. W. Seeley, B. L. Miller, A. M. Karydas, H. Rosen, E. G. P. Dopper, H. Seelaar, G. Logroscino, R. Capozzo, V. Novelli, A. A. Puca, M. Franceschi, A. Postiglione, G. Milan, P. Sorrentino, M. Kristiansen, H.-H. Chiang, C. Graff, F. Pasquier, A. Rollin, V. Deramecourt, T. Lebouvier, D. Kapogiannis, L. Ferrucci, S. Pickering-Brown, A. B. Singleton, J. Hardy, P. Momeni, RiMod-FTD (Risk and Modifying factors in Fronto-Temporal Dementia), Netherlands Brain Bank (NBB), Bradley F. Boeve, Ronald C. Petersen, Tanis J. Ferman, Jay A. van Gerpen, Marcel J. T. Reinders, Ryan J. Uitti, Lluís Tárraga, Wolfgang Maier, Oriol Dols-Icardo, Amit Kawalia, Maria Carolina Dalmasso, Mercè Boada, Uwe K. Zettl, Natasja M. van Schoor, Marian Beekman, Mariet Allen, Eliezer Masliah, Adolfo López de Munain, Alexander Pantelyat, Zbigniew K. Wszolek, Owen A. Ross, Dennis W. Dickson, Neill R. Graff-Radford, David Knopman, Rosa Rademakers, Afina W. Lemstra, Yolande A. L. Pijnenburg, Philip Scheltens, Thomas Gasser, Patrick F Chinnery, Bernhard Hemmer, Martijn A. Huisman, Juan Troncoso, Fermin Moreno, Ellen A. Nohr, Thorkild I. A. Sørensen, Peter Heutink, Pascual Sánchez-Juan, Danielle Posthuma, The GIFT (Genetic Investigation in Frontotemporal Dementia and Alzheimer’s Disease) Study Group, G. Coppola, A. M. Karydas, A. Varpetian, T. M. Foroud, A. I. Levey, W. A. Kukull, M. F. Mendez, J. Ringman, H. Chui, C. Cotman, C. DeCarli, B. L. Miller, D. H. Geschwind, Jordi Clarimón, Kaare Christensen, Nilüfer Ertekin-Taner, Sonja W. Scholz, Alfredo Ramirez, Agustín Ruiz, Eline Slagboom, Wiesje M. van der Flier, Henne Holstege

**Affiliations:** 1 Alzheimer Center Amsterdam, Department of Neurology, Amsterdam Neuroscience, Vrije Universiteit Amsterdam, Amsterdam UMC, Amsterdam, The Netherlands; 2 Department of Clinical Genetics, Vrije Universiteit Amsterdam, Amsterdam UMC, Amsterdam, The Netherlands; 3 Department of Neuroscience, Mayo Clinic Florida, Jacksonville, FL 32224 USA; 4 Department of Complex Trait Genetics, Center for Neurogenomics and Cognitive Research, Amsterdam Neuroscience, Vrije Universiteit Amsterdam, Amsterdam UMC, Amsterdam, The Netherlands; 5 Department for Neurodegenerative Diseases and Geriatric Psychiatry, University of Bonn, Bonn, Germany; 6 DZNE, German Center for Neurodegenerative Diseases, Bonn, Germany; 7 Division of Neurogenetics and Molecular Psychiatry, Department of Psychiatry and Psychotherapy, Faculty of Medicine, University Hospital Cologne, Cologne, Germany; 8 Molecular Epidemiology, Leiden University Medical Center, Leiden, The Netherlands; 9 Pattern Recognition and Bioinformatics, Delft University of Technology, Delft, The Netherlands; 10 Research Center and Memory Clinic, Fundació ACE, Institut Català de Neurociències Aplicades, Universitat Internacional de Catalunya, Barcelona, Spain; 11 Centro de Investigacion Biomedica en Red en Enfermedades Neurodegenerativas (CIBERNED), Madrid, Spain; 12 Department of Neurology, Brain Center Rudolf Magnus, University Medical Center Utrecht, Utrecht, The Netherlands; 13 Amsterdam UMC-Vrije Universiteit Amsterdam, Department of Epidemiology and Biostatistics, Amsterdam Public Health Research Institute, Amsterdam, The Netherlands; 14 Interdepartmental Program in Bioinformatics, University of California, Los Angeles, USA; 15 Neuropsychiatric Epidemiology Unit, Department of Psychiatry and Neurochemistry, Institute of Neuroscience and Physiology, Sahlgrenska Academy, Centre for Ageing and Health (AgeCap) at the University of Gothenburg, Gothenburg, Sweden; 16 Max Planck Institute of Psychiatry, Munich, Germany; 17 Department of Neurology, Klinikum rechts der Isar, Technical University of Munich, Munich, Germany; 18 German Competence Network Multiple Sclerosis (KKNMS), Munich, Germany; 19 Movement Disorders and Memory Unit, Department of Neurology, University Hospital Mutua de Terrassa, Barcelona, Spain; 20 Fundacio per la Recerca Biomedica I Social Mutua Terrassa, Terrassa, Barcelona Spain; 21 German Center for Neurodegenerative Diseases (DZNE)-Tübingen, Tübingen, Germany; 22 Hertie Institute for Clinical Brain Research, University of Tübingen, Tübingen, Germany; 23 Memory Unit, Department of Neurology, IIB Sant Pau, Hospital de la Santa Creu i Sant Pau, Universitat Autonoma de Barcelona, Barcelona, Spain; 24 Clinical Neurochemistry Laboratory, Sahlgrenska University Hospital, Mölndal, Sweden; 25 Department of Psychiatry and Neurochemistry, Institute of Neuroscience and Physiology, Sahlgrenska Academy at the University of Gothenburg, Gothenburg, Sweden; 26 Department of Molecular Neuroscience, UCL Institute of Neurology, Queen Square, London, UK; 27 The Danish Aging Research Center, Epidemiology, Biostatistics and Biodemography, Department of Public Health, University of Southern Denmark, Odense, Denmark; 28 Neurodegenerative Diseases Research Unit, National Institute of Neurological Disorders and Stroke, Bethesda, MD 20892-3707 USA; 29 Epidemiology, Biostatistics and Biodemography, Department of Public Health, University of Southern Denmark, Odense, Denmark; 30 Institute of Genetic Medicine, Newcastle University, Newcastle upon Tyne, NE1 3BZ UK; 31 Department of Clinical Neurosciences, University of Cambridge, Cambridge, CB2 0QQ UK; 32 Institut für Neuroimmunologie und Multiple Sklerose (INIMS), Universitätsklinikum Hamburg‐Eppendorf, Hamburg, Germany; 33 Department of Pathology (Neuropathology), Johns Hopkins University Medical Center, Baltimore, MD USA; 34 Instituto Biodonostia, San Sebastian, Spain; 35 University Hospital “Marques de Valdecilla”, Santander, Spain; 36 IDIVAL, Santander, Spain; 37 Institute of Social Medicine, Occupational Health and Public Health (ISAP), University of Leipzig, Leipzig, Germany; 38 Department of Neurology, University of Leipzig, Leipzig, Germany; 39 Newcastle Brain Tissue Resource, Edwardson Building, Institute of Neuroscience, Newcastle University, Newcastle upon Tyne, NE4 5PL UK; 40 Cognitive Disorders Unit, Department of Neurology, Hospital Universitario San Sebastian, San Sebastian, Spain; 41 Department of Radiology and Nuclear Medicine, Amsterdam Neuroscience, Vrije Universiteit Amsterdam, Amsterdam UMC, Amsterdam, The Netherlands; 42 Department of Primary Medical Care, Center for Psychosocial Medicine, University Medical Center, Hamburg-Eppendorf, Germany; 43 Centre for Clinical Brain Sciences, University of Edinburgh, Edinburgh, EH4 2XU UK; 44 Department of Neurology, Klinik für Neurologie mit Institut für Translationale Neurologie, University of Münster, Münster, Germany; 45 Department of Neurology, Mayo Clinic Minnesota, Rochester, MN 55905 USA; 46 Department of Psychiatry and Psychology, Mayo Clinic Florida, Jacksonville, FL 32224 USA; 47 Department of Neurology, Mayo Clinic Florida, Jacksonville, FL 32224 USA; 48 Fundación Instituto Leloir-IIBBA-CONICET, Buenos Aires, Argentina; 49 Department of Neurology, University of Rostock, Rostock, Germany; 50 Laboratory of Neurogenetics, National Institute on Aging, National Institutes of Health, Bethesda, MD USA; 51 Department of Neurology, Hospital Universitario San Sebastian, San Sebastian, Spain; 52 Department of Neurology, Johns Hopkins University Medical Center, Baltimore, MD 21287 USA; 53 Center of Neurology, Department of Neurodegenerative diseases, Hertie-Institute for Clinical Brain Research, University of Tuebingen, Tuebingen, Germany; 54 MRC Mitochondrial Biology Unit, University of Cambridge, Cambridge, CB2 0QQ UK; 55 Munich Cluster for Systems Neurology (SyNergy), Munich, Germany; 56 Department of Sociology, VU University, Amsterdam, The Netherlands; 57 Research Unit of Gynecology and Obstetrics, Department of Clinical Research, University of Southern Denmark, Odense, Denmark; 58 Novo Nordisk Foundation Center for Basic Metabolic Research, Section of Metabolic Genetics, Copenhagen, Denmark; 59 Department of Public Health, Section of Epidemiology, Faculty of Health and Medical Sciences, University of Copenhagen, Copenhagen, Denmark; 60 MRC Integrative Epidemiology Unit, Bristol University, Bristol, UK; 61 Clinical Biochemistry and Pharmacology, Odense University Hospital, Odense, Denmark; 62 Department of Clinical Genetics, Odense University Hospital, Odense, Denmark; 63 Dutch Society for Research on Ageing, Leiden, The Netherlands; 64 Delft Bioinformatics Lab, Delft University of Technology, Delft, The Netherlands

## Correction to: Acta Neuropathologica (2019) 138:237–250 10.1007/s00401-019-02026-8

The IPDGC (The International Parkinson Disease Genomics Consortium) and EADB (Alzheimer Disease European DNA biobank) are listed correctly as an author to the article, however, they were incorrectly listed more than once.

The following should be added to the Acknowledgements:

The authors wish to thank the following for their contributors to IFGC: S. Mead; M. Synofzik; J. C. van Swieten; I. Leber; R. Ferrari; D. G. Hernandez; M. A. Nalls; J. D. Rohrer; A. Ramasamy; J. B. J. Kwok; C. Dobson-Stone; P. R. Schofield; G. M. Halliday; J. R. Hodges; O. Piguet; L. Bartley; E. Thompson; B. Borroni; A. Padovani; C. Cruchaga; N. J. Cairns; L. Benussi; G. Binetti; R. Ghidoni; G. Forloni; D. Albani; D. Galimberti; C. Fenoglio; M. Serpente; E. Scarpini; R. Blesa; M. Landqvist Waldö; K. Nilsson; C. Nilsson; I. R. A. Mackenzie; G.-Y. R. Hsiung; D. M. A. Mann; J. Grafman; C. M. Morris; J. Attems; T. D. Griffiths; I. G. McKeith; A. J. Thomas; P. Pietrini; E. D. Huey; E. M. Wassermann; A. Baborie; E. Jaros; M. C. Tierney; C. Razquin; S. Ortega-Cubero; E. Alonso; R. Perneczky; J. Diehl-Schmid; P. Alexopoulos; A. Kurz; I. Rainero; E. Rubino; L. Pinessi; E. Rogaeva; P. St George-Hyslop; G. Rossi; F. Tagliavini; G. Giaccone; J. B. Rowe; J. C. M. Schlachetzki; J. Uphill; J. Collinge; A. Danek; V. M. Van Deerlin; M. Grossman; J. Q. Trojanowski; J. van der Zee; C. Van Broeckhoven; S. F. Cappa; D. Hannequin; V. Golfier; M. Vercelletto; A. Brice; B. Nacmias; S. Sorbi; S. Bagnoli; I. Piaceri; J. E. Nielsen; L. E. Hjermind; M. Riemenschneider; M. Mayhaus; B. Ibach; G. Gasparoni; S. Pichler; W. Gu; M. N. Rossor; N. C. Fox; J. D. Warren; M. G. Spillantini; H. R. Morris; P. Rizzu; J. S. Snowden; S. Rollinson; A. Richardson; A. Gerhard; A. C. Bruni; R. Maletta; F. Frangipane; C. Cupidi; L. Bernardi; M. Anfossi; M. Gallo; M. E. Conidi; N. Smirne; M. Baker; K. A. Josephs; J. E. Parisi; W. W. Seeley; B. L. Miller; A. M. Karydas; H. Rosen; E.G. P. Dopper; H. Seelaar; G. Logroscino; R. Capozzo; V. Novelli; A. A. Puca; M. Franceschi; A. Postiglione; G. Milan; P. Sorrentino; M. Kristiansen; H.-H. Chiang; C. Graff; F. Pasquier; A. Rollin; V. Deramecourt; T. Lebouvier; D. Kapogiannis; L. Ferrucci; S. Pickering-Brown; A. B. Singleton; J. Hardy; P. Momeni.

This work was supported in part by RiMod-FTD, an EU Joint Programme-Neurodegenerative Disease Research (JPND) project. We acknowledge the following contributors to RiMoD-FTD: John van Swieten (Erasmus University Medical Center, Rotterdam, the Netherlands); Simon Mead (UCL Institute of Prion Diseases, London, UK); Isabel LeBer (Sorbonne Universités, UPMC Univ Paris 06, Inserm U1127, CNRS UMR 7225, Institut du Cerveau et la Moelle épinière (ICM), Hôpital Pitié-Salpêtrière, Paris, France); Matthis Synofzik (Department of Neurodegenerative Diseases, Center for Neurology and Hertie-Institute for Clinical Brain Research, University of Tübingen and German Center for Neurodegenerative Diseases (DZNE), Tübingen, Germany).

