# Supplementary material for: A nonsynonymous mutation in PLCG2 reduces the risk of Alzheimer’s disease, dementia with Lewy bodies and frontotemporal dementia, and increases the likelihood of longevity
Source: Acta Neuropathol. 2019 May 26;138(2):237–50. doi: 10.1007/s00401-019-02026-8 (PMC6660501; doi:10.1007/s00401-019-02026-8)
Supplement: Supplementary file 2 — Supplementary material 2 (PDF 972 kb) [file 401_2019_2026_MOESM2_ESM.pdf]

Content: Supplementary Figures 1-10

Cohort	Dementia	Age-difference
UCLA/UCSF GIFT	FTD	-10.9
Swedish studies	AD	-7.1
Spanish cohorts	PSP	-3.9
Brain compendium	FTD	-3.7
Spanish cohorts	AD	-2.4
Spanish cohorts	FTD	-0.6
UCLA/UCSF GIFT	AD	0
Brain compendium	DLB	0.8
Dutch cohorts	FTD	1.6
Brain compendium	AD	2.3
Spanish cohorts	DLB	2.8
UCLA/UCSF GIFT	PSP	4
Mayo Clinic	PD	4
Brain compendium	PSP	5.5
Dutch cohorts	AD	7.3
Dutch cohorts	DLB	7.3
Mayo Clinic	DLB	14.2
Mayo Clinic	PSP	16.2
Mayo Clinic	AD	21.8

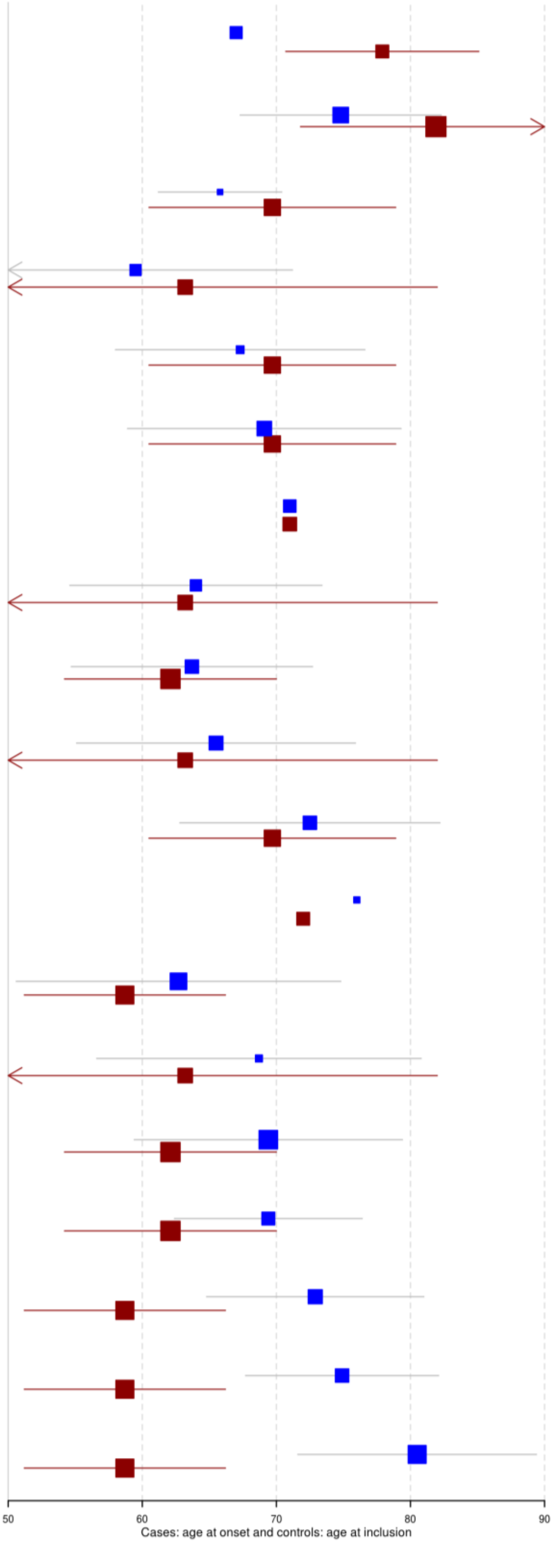

Supplementary Figure 1: Average age of cases (blue) and controls (red) in cohorts. Wiskers show the standard deviation of the average age. Cohorts are ordered by the difference in age between cases and controls. Most cohort have controls of the same age or lower than the cases.

AD

Study	N-cases	N-controls	MAF-cases	MAF-controls	Odds-ratio	p-value
UCLA/UCSF GIFT	224	249	0.00	2.01	0.00 [0.00-Inf]	0.982
NDRU cohort	527	343	0.66	1.17	0.56 [0.20-1.57]	0.272
Brain compendium	277	362	0.00	0.83	0.00 [0.00-Inf]	0.979
Spanish cohorts	23	746	0.00	0.67	0.00 [0.00-Inf]	0.991
Swedish studies	564	3480	0.53	0.88	0.60 [0.26-1.40]	0.239
MAYO	1477	1487	0.64	0.98	0.66 [0.37-1.17]	0.155
Amsterdam UMC	1893	2571	0.63	1.24	0.50 [0.31-0.81]	0.004
**Combined OR**	**4985**	**9238**	**0.56**	**1.02**	**0.57 [0.41-0.78]**	**0.000**

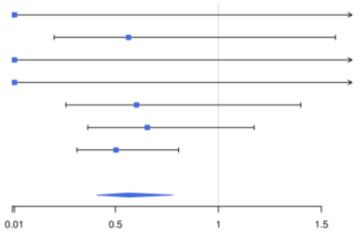

Supplementary Figure 2: Alzheimer's Disease (AD) cohort-specific associations of rs72824905-G. Numbers (N) of AD patients and controls studied. MAF = minor allele frequency. The figure shows the odds-ratio (box) of the rs72824905-G with the 95% confidence intervals (whiskers) per study. The combined OR and 95% confidence interval is shown as a triangle.

DLB

Study	N-cases	N-controls	MAF-cases	MAF-controls	Odds-ratio	p-value
NDRU cohort	622	343	0.64	1.17	0.55 [0.20-1.47]	0.230
Brain compendium	97	362	0.52	0.83	0.62 [0.07-5.20]	0.658
Spanish cohorts	232	746	0.65	0.67	0.96 [0.26-3.53]	0.956
MAYO	306	1487	0.33	0.98	0.33 [0.08-1.39]	0.132
Amsterdam UMC	189	2571	0.53	1.24	0.42 [0.10-1.73]	0.228
**Combined OR**	**1446**	**5509**	**0.55**	**1.06**	**0.54 [0.30-0.99]**	**0.045**

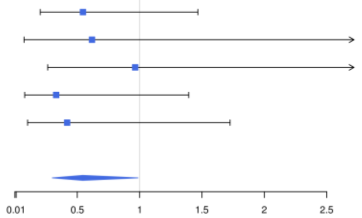

Supplementary Figure 3: Dementia with Lewy-bodies (DLB) cohort-specific associations of rs72824905-G. Numbers (N) of DLB patients and controls studied. MAF = minor allele frequency. The figure shows the odds-ratio (box) of the rs72824905-G with the 95% confidence intervals (whiskers) per study. The combined OR and 95% confidence interval is shown as a triangle.

FTD

Study	N-cases	N-controls	MAF-cases	MAF-controls	Odds-ratio	p-value
RiMod-FTD	255	1660	0.59	1.17	0.51 [0.16-1.63]	0.254
IFGC	1360	5059	0.81	1.17	0.69 [0.44-1.09]	0.111
UCLA/UCSF GIFT	132	249	0.76	2.01	0.41 [0.09-1.80]	0.239
Brain compendium	93	362	1.08	0.83	1.30 [0.26-6.57]	0.748
Spanish cohorts	366	746	0.14	0.67	0.20 [0.03-1.58]	0.127
Amsterdam UMC	231	2571	0.22	1.24	0.17 [0.02-1.23]	0.080
**Combined OR**	**2437**	**10647**	**0.64**	**1.16**	**0.61 [0.41-0.89]**	**0.011**

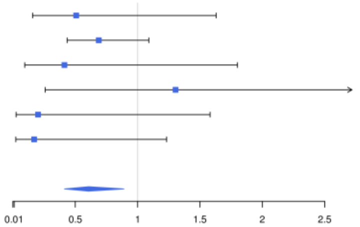

Supplementary Figure 4: Frontal Temporal Dementia (FTD) cohort-specific associations of rs72824905-G. Numbers (N) of FTD patients and controls studied. MAF = minor allele frequency. The figure shows the odds-ratio (box) of the rs72824905-G with the 95% confidence intervals (whiskers) per study. The combined OR and 95% confidence interval is shown as a triangle.

PSP

Study	N-cases	N-controls	MAF-cases	MAF-controls	Odds-ratio	p-value
UCLA/UCSF GIFT	12	249	0.00	2.01	0.00 [0.00-Inf]	0.991
NDRU cohort	613	343	0.90	1.17	0.77 [0.30-1.92]	0.569
Brain compendium	17	362	2.94	0.83	3.71 [0.42-32.66]	0.238
Spanish cohorts	9	746	0.00	0.67	0.00 [0.00-Inf]	0.992
MAYO	231	1487	1.95	0.98	2.04 [0.95-4.36]	0.067
**Combined OR**	**882**	**3187**	**1.19**	**0.99**	**1.46 [0.83-2.58]**	**0.186**

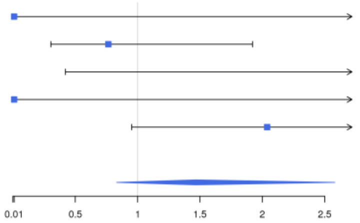

Supplementary Figure 5: Progressive Supranuclear Palsy (PSP) cohort-specific associations of rs72824905-G. Numbers (N) of PSP patients and controls studied. MAF = minor allele frequency. The figure shows the odds-ratio (box) of the rs72824905-G with the 95% confidence intervals (whiskers) per study. The combined OR and 95% confidence interval is shown as a triangle.

ALS

Study	N-cases	N-controls	MAF-cases	MAF-controls	Odds-ratio	p-value
stratum s1	423	420	0.84	0.74	1.15 [0.36-3.68]	0.8170
stratum s2	299	317	1.25	0.76	1.86 [0.52-6.69]	0.3324
stratum s3	145	4882	1.29	1.03	1.44 [0.48-4.36]	0.5347
stratum s4	288	268	0.48	0.41	0.81 [0.12-5.71]	0.8363
stratum s6	168	159	0.81	1.32	0.56 [0.10-2.97]	0.4859
stratum s8	308	331	0.50	0.57	0.85 [0.16-4.41]	0.8452
stratum s9	614	2687	1.34	0.78	1.73 [0.95-3.16]	0.0846
stratum s10	266	513	0.66	0.95	0.65 [0.17-2.46]	0.5102
stratum s11	382	244	1.42	0.28	10.48 [1.11-98.64]	0.0080
stratum s13	952	1829	0.70	0.97	0.73 [0.38-1.41]	0.3465
stratum s14	518	258	0.65	1.39	0.42 [0.14-1.30]	0.1333
stratum s15	290	93	1.04	1.57	0.60 [0.13-2.86]	0.5346
stratum s17	203	221	0.89	1.50	0.42 [0.09-1.84]	0.2379
stratum s18	205	242	0.42	1.04	0.35 [0.05-2.32]	0.2457
stratum s21	264	443	0.02	0.87	0.00 [0.00-550327.04]	0.0034
stratum s22	559	2003	0.71	0.87	0.81 [0.35-1.84]	0.5987
stratum s23	327	1005	0.93	1.21	0.68 [0.26-1.81]	0.4279
stratum s24	1032	2502	0.81	0.73	1.14 [0.61-2.12]	0.6850
stratum s25	1399	648	1.05	0.94	0.96 [0.39-2.34]	0.9229
stratum s26	1715	1075	0.82	1.19	0.62 [0.35-1.12]	0.1184
stratum s27	596	533	1.43	1.18	1.22 [0.59-2.51]	0.5950
**Combined OR**	**10953**	**20673**	**0.89**	**0.93**	**1.07 [0.87-1.33]**	**0.5187**

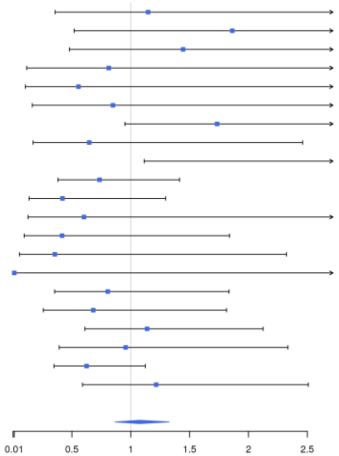

Supplementary Figure 6: Amyotrophic Lateral Sclerosis (ALS) **c**ohort-specific associations of rs72824905-G. Numbers (N) of ALS patients and controls studied. MAF = minor allele frequency. The figure shows the odds-ratio (box) of the rs72824905-G with the 95% confidence intervals (whiskers) per study. The combined OR and 95% confidence interval is shown as a triangle.

PD

Study	N-cases	N-controls	MAF-cases	MAF-controls	Odds-ratio	p-value
MAYO clinic PD	853	1487	1.06	0.98	1.08 [0.60-1.96]	0.791
IPDGC	27595	106951			1.20 [0.97-1.48]	0.094
**Combined OR**	**28448**	**108438**			**1.18 [0.97-1.44]**	**0.095**

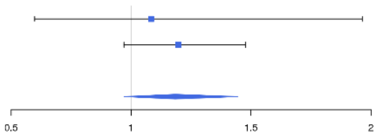

Supplementary Figure 7: Parkinson's Disease (PD) cohort-specific associations of rs72824905-G. Numbers (N) of PD patients and controls studied. MAF = minor allele frequency. The figure shows the odds-ratio (box) of the rs72824905-G with the 95% confidence intervals (whiskers) per study. The combined OR and 95% confidence interval is shown as a triangle.

Longevity

Study	N-cases	N-controls	MAF-cases	MAF-controls	Odds-ratio	p-value
GBC	770	2709	1.04	0.83	1.26 [0.71-2.24]	0.438
LLS	1138	743	1.23	0.74	1.68 [0.83-3.39]	0.149
AgeCoDe	462	861	1.52	1.16	1.31 [0.66-2.63]	0.440
Danish studies	853	2793	0.59	0.59	0.99 [0.49-2.02]	0.983
Amsterdam UMC	293	2571	2.73	1.24	2.26 [1.29-3.97]	0.004
**Combined OR**	**3516**	**9677**	**1.19**	**0.89**	**1.49 [1.12-1.98]**	**0.006**

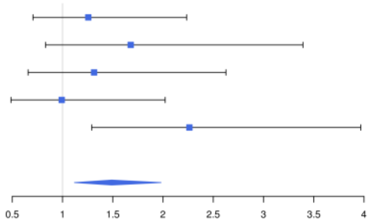

Supplementary Figure 8: Longevity cohort-specific associations of rs72824905-G. Numbers (N) of long-lived individuals and controls studied. MAF = minor allele frequency. The figure shows the odds-ratio (box) of the rs72824905-G with the 95% confidence intervals (whiskers) per study. The combined OR and 95% confidence interval is shown as a triangle.

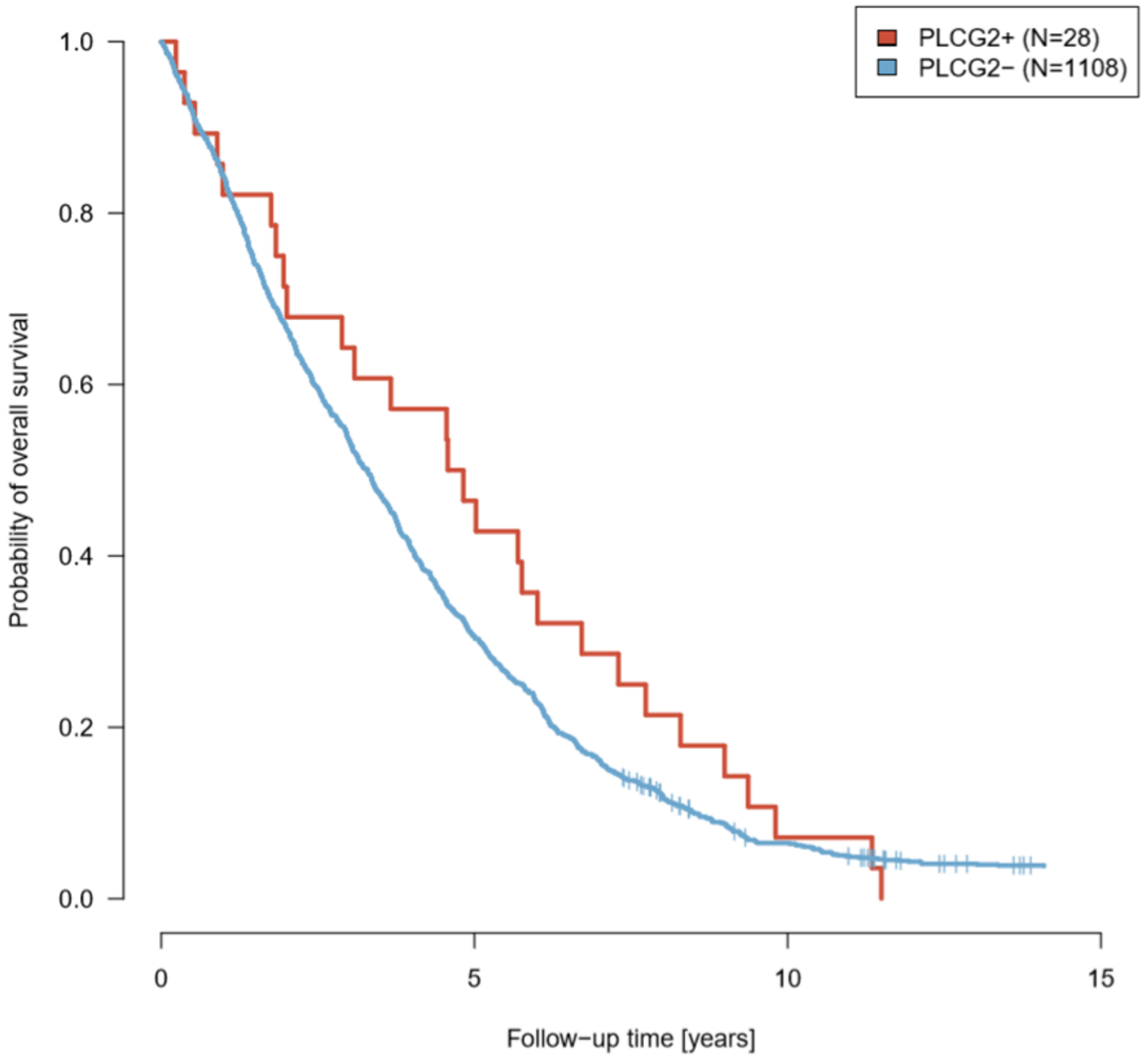

Supplementary Figure 9: Survival after the age of 90 years of rs72824905-G carriers vs. non-carriers. A Kaplan-Meier curves are shown. Whiskers show individuals that were censored.

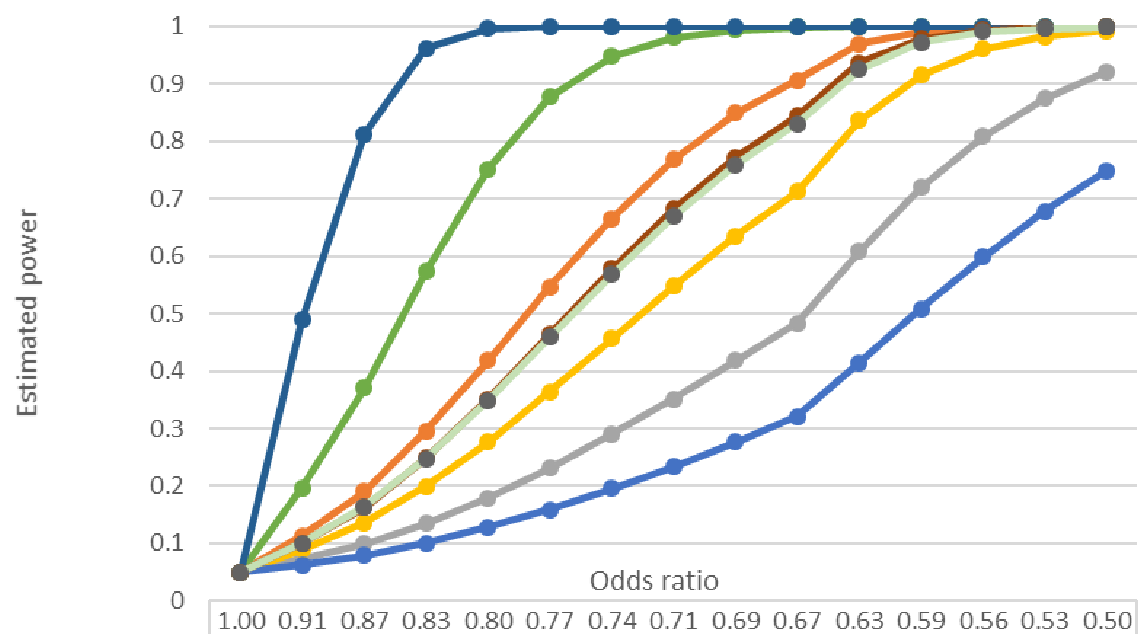

	1.00	0.91	0.87	0.83	0.80	0.77	0.74	0.71	0.69	0.67	0.63	0.59	0.56	0.53	0.50
AD	0.05	0.11	0.19	0.3	0.42	0.55	0.67	0.77	0.85	0.91	0.97	0.99	1	1	1
DLB	0.05	0.07	0.1	0.14	0.18	0.23	0.29	0.35	0.42	0.48	0.61	0.72	0.81	0.88	0.92
FTD	0.05	0.09	0.14	0.2	0.28	0.36	0.46	0.55	0.64	0.71	0.84	0.92	0.96	0.98	0.99
PSP	0.05	0.06	0.08	0.1	0.13	0.16	0.2	0.23	0.28	0.32	0.42	0.51	0.6	0.68	0.75
ALS	0.05	0.2	0.37	0.57	0.75	0.88	0.95	0.98	0.99	1	1	1	1	1	1
PD	0.05	0.49	0.81	0.96	1	1	1	1	1	1	1	1	1	1	1
MS	0.05	0.1	0.16	0.25	0.35	0.46	0.58	0.68	0.77	0.84	0.94	0.98	0.99	1	1
Longevity	0.05	0.1	0.16	0.25	0.35	0.46	0.57	0.67	0.76	0.83	0.93	0.97	0.99	1	1

Supplementary figure 10: Power analysis for Alzheimer’s disease (AD), Frontotemporal Dementia (FTD), dementia with Lewy-bodies (DLB), Progressive Supranuclear Palsy (PSP), Parkinson’s Disease (PD) Amyotrophic Lateral Sclerosis (ALS) and Multiple Sclerosis (MS). Statistical power of our analysis to attain a *p*-value of 0.05 is shown for the odds ratios (ORs) between 0.50 and 1. We used the total number of cases and controls from our analysis. We assumed an additive model, a minor allele frequency of 0.009 and a disease frequency of 0.01 for all diseases. The table shows the exact estimates of statistical power.
